# Impact of structural dynamics on biological functions of flaviviruses

**DOI:** 10.1111/febs.16419

**Published:** 2022-03-11

**Authors:** Karin Stiasny, Iris Medits, Lena Roßbacher, Franz X. Heinz

**Affiliations:** ^1^ 27271 Center for Virology Medical University of Vienna Austria

**Keywords:** antibody‐dependent enhancement, envelope protein, flavivirus, membrane fusion, particle dynamics, particle heterogeneity, tick‐borne encephalitis virus, virus breathing

## Abstract

Flaviviruses comprise a number of mosquito‐ or tick‐transmitted human pathogens of global public health importance. Advances in structural biology techniques have contributed substantially to our current understanding of the life cycle of these small enveloped RNA viruses and led to deep insights into details of virus assembly, maturation and cell entry. In addition to large‐scale conformational changes and oligomeric rearrangements of envelope proteins during these processes, there is increasing evidence that smaller‐scale protein dynamics (referred to as virus “breathing”) can confer extra flexibility to these viruses for the fine‐tuning of their interactions with the immune system and possibly with cellular factors they encounter in their complex ecological cycles in arthropod and vertebrate hosts. In this review, we discuss how work with tick‐borne encephalitis virus has extended our view on flavivirus breathing, leading to the identification of a novel mechanism of antibody‐mediated infection enhancement and demonstrating breathing intermediates of the envelope protein in the process of membrane fusion. These data are discussed in the context of other flaviviruses and the perspective of a potential role of virus breathing to cope with the requirements of adaptation and replication in evolutionarily very different hosts.

## Graphical abstract

Small‐scale dynamics of the major envelope protein E of flaviviruses (“virus breathing”) together with binding of specific antibodies can promote fusion‐loop exposure and insertion into plasma membranes of target cells, providing the basis for a special mechanism of antibody‐mediated enhanced infection. Upon cellular uptake by endocytosis, dynamic “breathing” intermediates are functionally important for the structural conversions of E into a post‐fusion conformation to drive membrane fusion in the endosome.

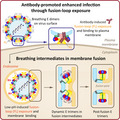

**Abbreviations**
ADE: antibody‐dependent enhancement
Cryo‐EM: cryogenic electron microscopy
DI: domain I
DII: domain II
DIII: domain III
ER: endoplasmic reticulum
FcγR: Fcγ receptors
FL: fusion loop
H3: helix 3
mab: monoclonal antibody
Pre‐F‐H1: pre‐fusion helix 1
Pre‐F‐H2: pre‐fusion helix 2
Pre‐F‐H3: pre‐fusion helix 3
TBE: tick‐borne encephalitis
TBEV: tick‐borne encephalitis virus
TGN: trans‐Golgi network
TM: transmembrane

## Introduction

Tick‐borne encephalitis virus (TBEV) is one of the important human‐pathogenic flaviviruses, which also comprise the mosquito‐borne yellow fever, dengue, West Nile, and Japanese encephalitis viruses, as well as the recently emerged Zika virus [1]. Flaviviruses are distributed globally and pose major public health problems, with dengue viruses (occurring in tropical and sub‐tropical regions) causing the highest disease incidence of all flaviviruses worldwide [2, 3]. In temperate zones, infections with TBEV result in > 10 000 hospitalized cases per year in its endemic regions of Europe and Asia, despite the availability of effective vaccines [4]. Clinically, the manifestations of flavivirus infections range from asymptomatic or mild febrile disease to haemorrhagic fevers (e.g. dengue, yellow fever) or severe neurological manifestations (e.g. TBE, Japanese encephalitis) [1]. Global warming due to climate change can promote the geographical expansion of arthropod vectors, thus leading to the appearance of arthropod‐borne viruses in previously unaffected areas [5]. As shown for TBEV, however, other incompletely understood factors, independent of the presence of the tick vector and animal virus reservoirs, apparently contribute to the control of the complex natural virus cycle and can lead to the emergence or decline of human disease in certain geographical regions [6, 7]. The requirement for transmission of flaviviruses by arthropod vectors implies adaptation of their cellular reproduction machinery to evolutionarily very distant hosts, which constrains their variability and includes adjustments to cellular dependency factors of both vertebrate and invertebrate host cells [8, 9, 10].

Despite the profoundly diverse clinical pictures caused by flaviviruses and transmission by different vectors, these small enveloped positive‐stranded RNA viruses are structurally very similar and have a common mode of assembly, mechanisms of maturation into infectious virions and cell entry [11]. During these processes, the major viral envelope protein E, which covers the surface of flaviviruses and plays a key role for virus entry, undergoes a number of structural changes that are controlled by differences of the pH in intracellular compartments as well as the extracellular fluid and interactions between E and a second viral envelope protein in immature virions (prM; see section “Conformational and oligomeric switches during assembly and maturation”) [11, 12]. This built‐in structural flexibility is not only required for oligomeric rearrangements and domain relocations in the viral life cycle, but the existing conformational space also endows the E protein with small‐scale oscillations [13, 14] that have been referred to as “virus breathing” [15]. Evidence is increasing that these oscillations can have a substantial impact on the biological properties of flaviviruses in different stages of the viral life cycle [12, 13, 14].

In this review, we describe the peculiar features of flavivirus assembly, maturation and entry as a structural basis for discussing two new aspects of E protein dynamics we recently identified in our work with TBEV, both of which are of significance for the processes of virus entry. The first describes a mechanism of antibody‐mediated conformational change of E that promotes interactions with target cells and enhances infection, and the second describes the importance of protein dynamics to sample structural intermediates of E in the process of membrane fusion.

## Structure of flaviviruses and virus entry

**Fig. 1:**
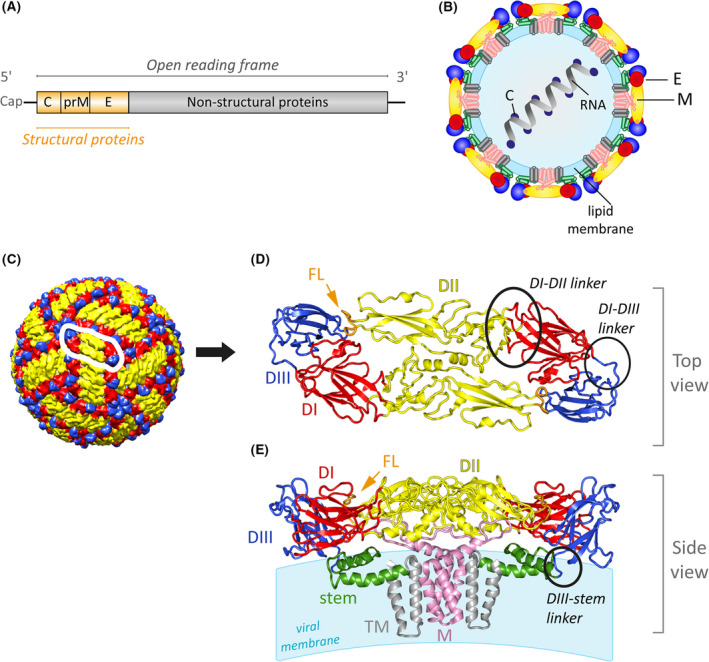
Structural organization of mature flaviviruses. (A) Flavivirus RNA genome with the single open reading frame. The three structural proteins are coloured yellow, the nonstructural proteins grey. (B) Schematic representation of a flavivirus particle in its fully mature state. (C) Cryo‐EM structure of the mature TBEV particle (PDB: 5O6A, [19]). One E dimer is encircled in white. (D) Top view of the TBEV E dimer ectodomain (without the stem‐anchor region) and (E) side view of the full‐length TBEV E‐M complex (PDB: 5O6A, [19]). The flexible linkers/hinges between the domains are encircled in black in one E monomer. The E protein is coloured according to its domains: domain I (DI), red; domain II (DII), yellow; domain III (DIII), blue; fusion loop (FL), orange; stem, green and transmembrane anchor (TM), grey. M is displayed in pink; the viral membrane in light blue. Molecular structures were generated with UCSF Chimera (panel C) (https://www.cgl.ucsf.edu/chimera, [93] and PyMol (panels D and E) (https://pymol.org/2).

Flaviviruses are small enveloped viruses (~ 50 nm diameter) with a positive‐strand RNA genome, encoding a single polyprotein that is co‐ and post‐translationally cleaved into three structural proteins [capsid (C), precursor of M (prM)/membrane (M), envelope (E)] and at least seven non‐structural proteins [11] (Fig. 1A). In mature virions, the M and E proteins are anchored in the host‐derived viral membrane that surrounds a nucleocapsid consisting of the RNA associated with the C protein (Fig. 1B). E is a bifunctional class II viral fusion protein, responsible for attachment to host cells as well as fusion of the viral with the endosomal membrane after uptake by receptor‐mediated endocytosis, resulting in the release of the viral genome into the cytoplasm [16, 17]. Because of its surface‐exposed location and key entry functions, E is the major inducer and target of neutralizing antibodies [18].

High‐resolution structures of mature virions have been determined by cryogenic electron microscopy (cryo‐EM) for several flaviviruses, including TBEV [19], all showing the same overall structural organization (reviewed in [1, 11]). 180 E proteins are arranged horizontally at the virion surface as 90 head‐to‐tail dimers in an icosahedral herringbone pattern (Fig. 1B‐E). The small M protein is buried under the E lattice and has almost no surface exposure. A high‐resolution structure of E was determined for TBEV by X‐ray crystallography already in 1995 [20], thus being the first description of a viral fusion protein now referred to as class II fusion proteins [17]. The E protein of all flaviviruses has a similar overall architecture. It is an elongated molecule containing three external domains (domains I, II, III; Fig. 1D,E) connected to a double membrane anchor by the so‐called stem region (Fig. 1E). The ectodomain possesses flexible linkers between domains I and II as well as domains I and III, which are important for structural changes and oligomeric rearrangements essential for assembly, maturation and entry. The stem is located between the E ectodomain and the viral membrane, connected to domain III via a flexible linker (Fig. 1E). The hydrophobic fusion loop at the tip of domain II is highly conserved across flavivirus E proteins and is crucial for initiating membrane fusion [14].

Flaviviruses enter host cells by receptor‐mediated endocytosis and probably use different receptors and/or attachment factors in different tissues of the various invertebrate and vertebrate hosts involved in their natural transmission cycles. Binding to host cells has been described to be heterogeneous and may involve plasma membrane proteins like integrins (reviewed in [21]), charged interactions of E with glycosaminoglycans (GAGs) or binding of carbohydrates on viral surface proteins to multiple C‐type lectins [1]. In addition, cellular lipid receptors (TIM/TAM) were shown to interact with the flavivirus lipid membrane (which is at least partially accessible in infectious particles) and mediate endocytosis, a process termed “apoptotic mimicry” [22].

**Fig. 2:**
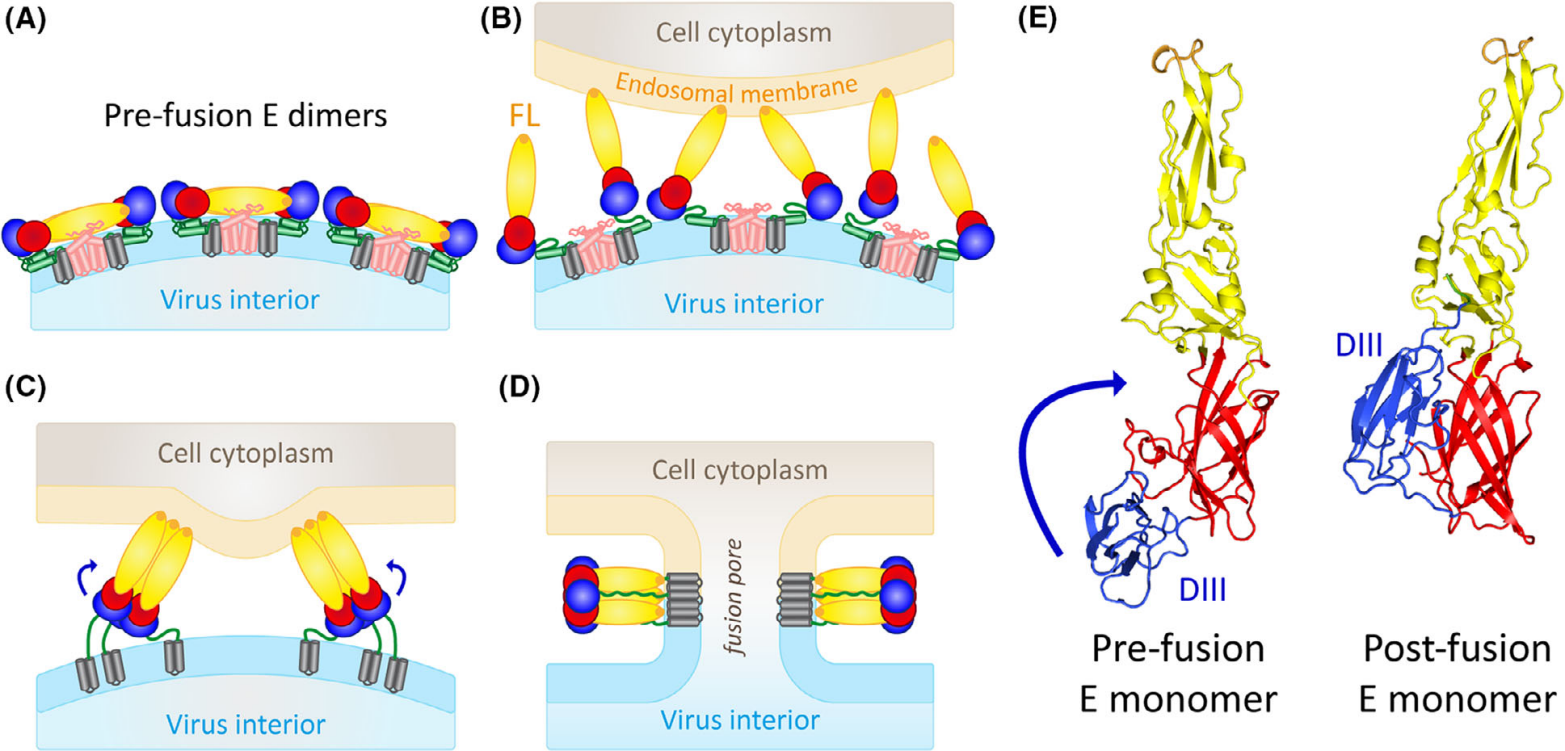
Flavivirus membrane fusion. (A) E dimers on the surface of the mature particle. (B) Low‐pH‐induced dissociation of the E dimers in endosomes, fusion‐loop exposure and insertion into the endosomal membrane. (C) Formation of trimers in an extended conformation bridging the two membranes, blue arrows indicate the subsequent relocation of domain III from the end to the side of the molecule (see also panel E). (D) Formation of the hairpin‐like post‐fusion trimer with the fusion loops and membrane anchors juxtaposed and opening of the fusion pore. (E) Ribbon representations of E monomers in their pre‐ and post‐fusion conformations, showing the relocation of domain III (DIII), indicated by a blue arrow in the pre‐fusion monomer. Colour code E: domain I, red; domain II, yellow; domain III, blue; fusion loop (FL), orange; stem, green and membrane anchor, grey. Molecular structures in panel E were generated with PyMol (https://pymol.org/2) using coordinates of TBEV (PDBs: 1SVB, 1URZ [20, 27]).

After cellular uptake, upon exposure of virions to the low pH in the endosome, inter‐ and intra‐molecular interactions in E as well as between E and M are weakened [23], leading to E dimer dissociation as a prerequisite to initiate fusion [24] (Fig. 2A). The now exposed fusion loops insert into the endosomal target membrane (Fig. 2B), and E homotrimers are formed with a head‐to‐head arrangement of the three subunits (Fig. 2C) [25]. As a next key step of the fusion process, the domains in the E subunits rearrange into a “hairpin”‐like conformation by the relocation of domain III, pulling the stem to the sides of a trimer core built by the domain I/II moieties [26, 27, 28, 29, 30, 31] (Fig. 2D). The reorganization of the pre‐fusion E dimer into the final hairpin‐like post‐fusion trimer, in which the fusion loops and membrane anchors are juxtaposed, catalyses the fusion of the two membranes, resulting in the opening of a fusion pore through which the genome is released into the cell cytoplasm (Fig. 2D) [16].

## Conformational and oligomeric switches during assembly and maturation

**Fig. 3:**
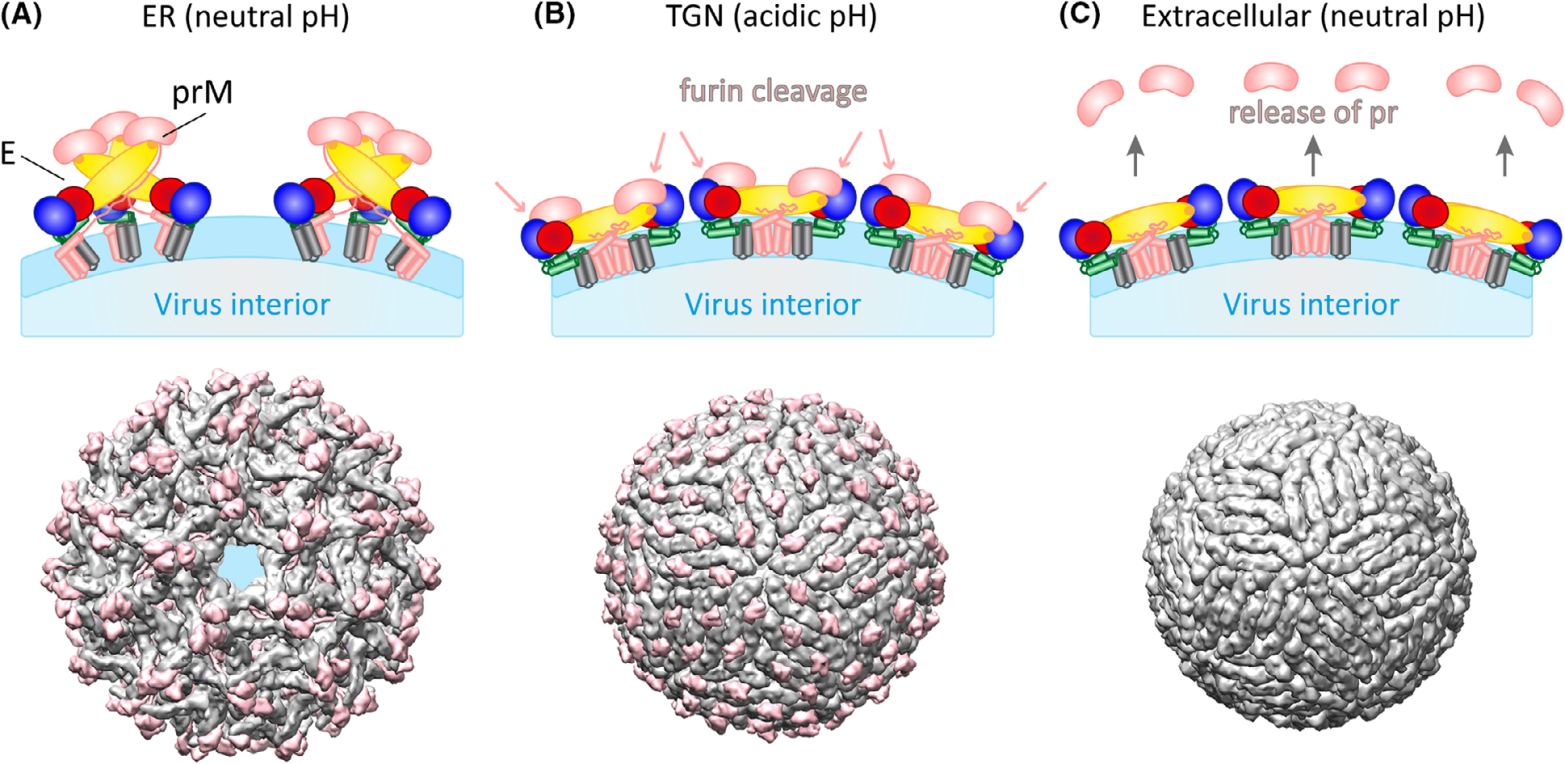
Rearrangements of viral surface proteins during maturation. Schematic representations in upper panels and cryo EM reconstructions in lower panels. (A) Immature virions assembled in the endoplasmic reticulum (ER) at neutral pH with trimeric prM/E spikes on the particle surface. (B) Formation of smooth‐surfaced particles with the herringbone‐like arrangement of E dimers induced by the acidic pH in the trans‐Golgi network (TGN) and cleavage of prM into pr and M. The cleavage product pr remains associated with the particles at acidic pH. (C) Mature infectious virion with the E dimers in a metastable conformation, primed for fusion. The cleaved pr parts have been released from the particles at neutral pH. Colour code of E and prM/M in the schematics as in Figs. 1 and 2. Molecular structures were generated with UCSF Chimera (https://www.cgl.ucsf.edu/chimera, [93]) using coordinates of dengue viruses (PDBs: 4B03, 3C6R, 4CCT; [34, 37]). E is shown in grey, prM and M in pink.

Flavivirus particles assemble by budding at membranes of the endoplasmic reticulum (ER), and are then transported through the secretory pathway to be released by exocytosis [11]. Since the compartments of the secretory pathway become progressively more acidic, with the trans‐Golgi network (TGN) reaching a pH value of ~ 6.0 [32], flaviviruses have evolved a complex assembly and maturation pathway to avoid premature fusion and hence inactivation during their transport [11]. prM plays a key role in these processes. It is co‐translated with E at ER‐associated ribosomes and acts as a chaperone for the proper folding of E [33]. The prM‐E heterodimers are the building blocks for ER budding of immature particles, which are non‐infectious and carry sixty trimeric spikes of prM/E heterodimers (Fig. 3A). High‐resolution structures of these first assembly products have been determined for several flaviviruses by cryo‐EM [34, 35, 36]. In these particles, the pr part of prM sits on top of the spikes, protecting the fusion loop of E from membrane interactions (Fig. 3A). During exocytotic transport, the acidic pH in the TGN triggers a complete reorganization of the spiky prM‐E trimers into a herringbone‐like lattice of head‐to‐tail E dimers associated with prM (Fig. 3B), resulting in smooth‐surfaced particles [37]. Through these rearrangements, prM exposes a protease‐cleavage site and is cleaved into pr and M by the TGN‐resident protease furin [37, 38]. Even after cleavage, pr remains bound to E at the acidic pH of the TGN (Fig. 3B) and thus prevents pre‐mature fusion at this stage of the viral life cycle [37, 39].

Upon particle secretion from the cell and encountering neutral pH, pr dissociates from E as a last step of virus maturation, thus generating infectious particles [37, 39] (Fig. 3C). In these mature particles, E exists in a metastable conformation, primed to undergo the low‐pH‐induced structural changes necessary for mediating viral fusion in endosomes, as described above (see Structure of flaviviruses and virus entry).

The maturation process can be incomplete, leading to the release of partially mature particles [40]. Especially with dengue viruses, furin cleavage has been reported to be less efficient than that of other flaviviruses due to a suboptimal cleavage site [41]. These resulting “mosaic” particles have their glycoproteins organized into two regions, one corresponding to the mature and the other to the immature structure, with an unstructured border zone between the two regions [42]. Such particles can be infectious, provided that the “mature patches” are sufficiently large to mediate fusion [43]. The extent of prM cleavage necessary for the transition from a non‐infectious immature virion to an infectious virus, however, is unknown [40]. There is evidence that fully immature particles can be proteolytically activated after entry into cells, when they are taken up by receptor‐mediated endocytosis and prM is cleaved by furin in endosomes [44].

## “Breathing” of flaviviruses

**Fig. 4:**
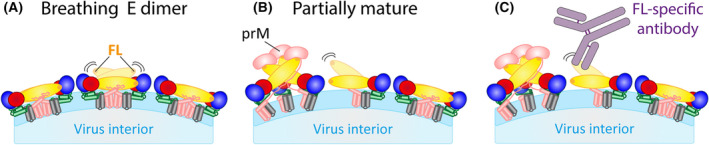
Schematic illustrations of structural heterogeneity of flavivirus particles. (A) E breathing (indicated by curved lines) of E on the surface of a mature virion, leading to the transient exposure of the fusion loop (FL). (B) Partially mature particles with a prM/E spike and E dimer representing the immature and mature patches on the virus surface, respectively. The border zone between the immature and mature regions (symbolized as a breathing E monomer) is irregular and therefore structurally not resolved. (C) Binding of a fusion‐loop (FL) specific antibody to E upon exposure of the fusion loop by breathing. Colour code of E and prM/M in the schematics as in Figs. 1 and 2.

The concept of flavivirus “breathing” was established by studies with monoclonal antibodies directed to seemingly inaccessible (cryptic) epitopes in the closed shell of E dimers at the surface of mature virions, which were nevertheless able to neutralize the virus (Fig. 1C) [45, 46, 47, 48, 49, 50, 51, 52, 53, 54, 55]. These data could only be explained by the transient exposure of otherwise buried surfaces of E, made possible by oscillations of E, most likely around the hinges that connect its domains (Fig. 1D,E) [13, 14, 56] (Fig. 4A). Several studies indicate that even single amino acid changes in E can impact the virion’s structural dynamics and influence the accessibility of buried epitopes and as a consequence the efficiency of virus neutralization [51, 52, 54].

Some prominent cryptic epitopes comprise the highly conserved fusion loop at the tip of domain II [14]. This sequence element is buried in the structures of fully mature flaviviruses as captured by cryo‐EM (Fig. 1C), but breathing around the domain I‐II hinge allows its transient exposure (Fig. 4A). Although antibodies recognizing the fusion loop are broadly cross‐reactive, their neutralizing potencies vary considerably among flaviviruses [45, 49, 57, 58, 59, 60, 61], with TBEV or Zika virus being rather resistant to neutralization compared to dengue viruses which are most sensitive [61].

Recently, studies with a conformation‐ and E dimer‐specific antibody (C10) that potently neutralizes both dengue and Zika viruses provided further evidence for substantial differences in the dynamics of flaviviruses and the effects of antibody binding [62, 63]. When bound to dengue virus serotype 2, the antibody caused increased dynamics and distortion of particles, whereas with Zika virus it reduced dynamics and stabilized the virus [62, 63]. Structural analyses of E protein‐C10 Fab complexes revealed an altered E dimer conformation of dengue serotypes 2, 3 and 4, in contrast to the high complementarity seen between C10 and the E dimer of Zika virus as well as dengue virus serotype 1 [62, 63, 64]. The obtained data thus indicate different modes of antibody recognition, although the effect was potent neutralization in all instances. Some of the authors therefore speculated that the dengue patients used as donors for C10‐antibody isolation had been primed by a prior Zika virus infection [63]. These and other experiments suggest that members of the dengue virus serocomplex are especially prone to breathing and the exposure of internal protein surfaces [13, 14]. Such a conclusion would also be consistent with the observation that dengue viruses (at least certain strains) are less thermostable than other flaviviruses [49, 58, 65, 66, 67] and readily change morphology when exposed to increased temperature [68, 69]. The biological consequences of this property are incompletely understood, but it certainly can influence virus neutralization.

In this context it is of note that incomplete maturation can also have an influence on the presentation of cryptic sites (including the fusion loop), presumably most pronounced at the unstructured border zone present in mosaic mature/immature particles (Fig. 4B,C) [13, 14] (see also “Conformational and oligomeric switches during assembly and maturation”). For instance, dengue virus taken directly from the plasma of infected people was more mature and resistant to neutralization by fusion‐loop‐specific antibodies than the same strain grown in cell culture [70].

## Structural dynamics and antibody‐mediated enhanced infection

**Fig. 5:**
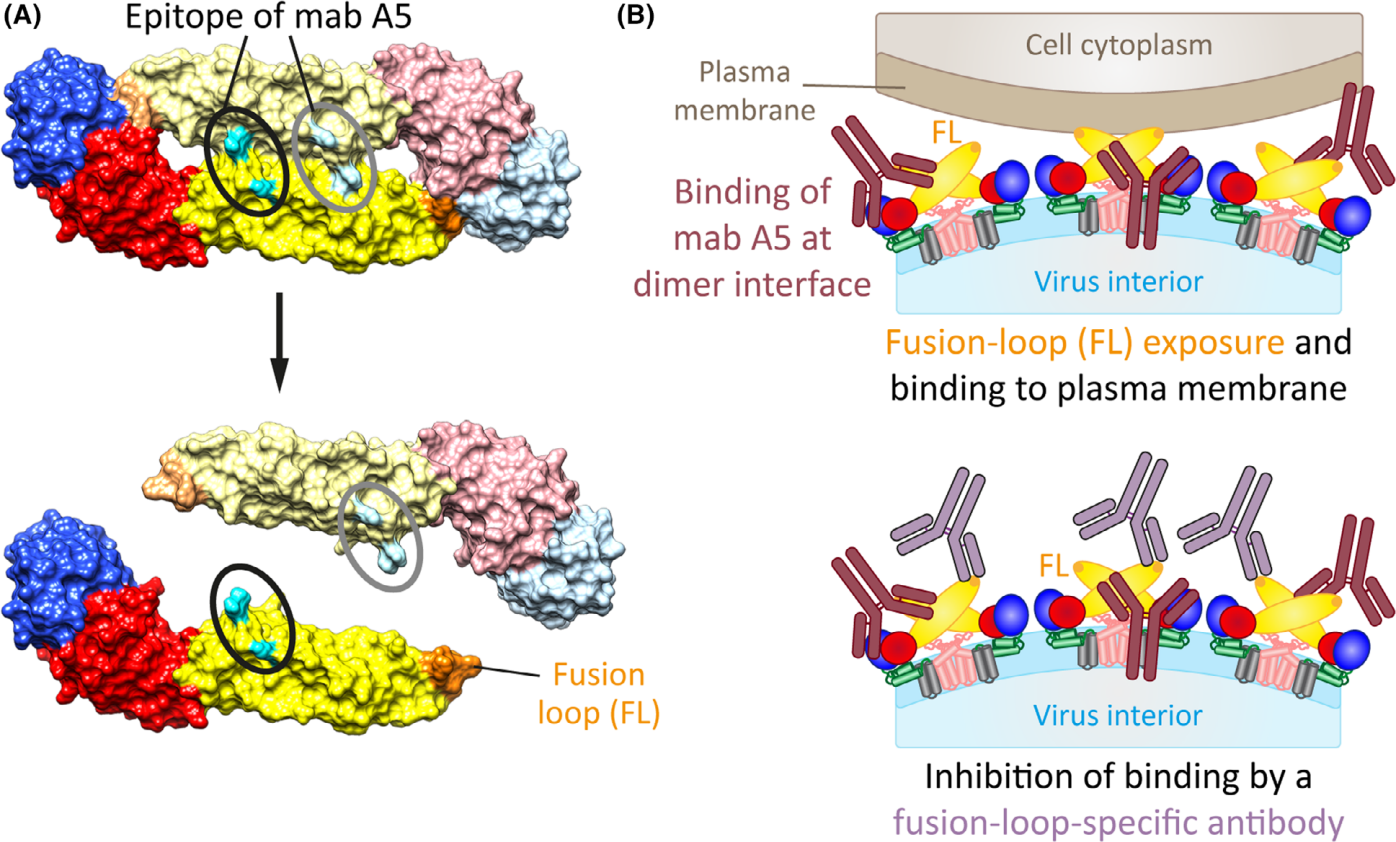
Promotion of TBEV cell binding and enhancement of infection induced by a monoclonal antibody (mab A5). (A) Surface representation of the TBEV E dimer (upper panel) and its dissociation into the two subunits by antibody A5. Amino acids contributing to the epitope of mab A5 (black and grey circles) are shown in cyan. (B) Schematic representation of antibody‐induced exposure of the fusion loop (FL), its attachment to the plasma membrane (upper panel) and inhibition of binding by a fusion‐loop‐specific antibody (lower panel). Colour code of E and pr/M as in Figs. 1 and 2. Molecular structures were generated with UCSF Chimera (https://www.cgl.ucsf.edu/chimera, [93]) using coordinates of the TBEV E dimer (PDB: 1SVB, [20]).

The structural dynamics of E molecules at the flavivirus surface may not only impact virus neutralization by antibodies, but can also cause antibody‐mediated effects, leading to enhanced infection. Such an effect was identified in experiments with TBEV [71] and shown to be unrelated to the long‐known phenomenon of antibody‐dependent enhancement of infection (ADE), resulting from the internalization of immune complexes into Fcγ‐receptor‐bearing cells. This phenomenon was described in many studies for several flaviviruses in vitro [72, 73] and linked to human disease in sequential dengue virus infections [74]. In contrast, the newly recognized mechanism of infection enhancement is based on the antibody‐induced exposure of the normally buried fusion loop, followed by its interaction with the plasma membrane of host cells and endocytosis of viral particles [71]. This mechanism was demonstrated with a monoclonal antibody (mab A5), which targets an epitope in domain II at the E dimer interface (Fig. 5A), and through its binding apparently shifts the equilibrium between monomeric and dimeric states of E, favouring the monomer with concomitant fusion‐loop exposure (Fig. 5A).

Specific inhibition experiments with a fusion‐loop specific antibody revealed that the mab A5‐exposed fusion loop was indeed responsible for direct virus binding to the plasma membrane (Fig. 5B), thereby increasing viral cell attachment and infectivity. Since antibody‐induced enhancement of binding was not only observed with cells but also with pure liposomes [71], it is likely that increased infection was due to fusion loop‐lipid interactions only and not to interactions with cellular plasma membrane proteins. Thus, the newly described ADE mechanism is based on the structural dynamics of E, antibody‐promoted conformational changes, and membrane interactions of the fusion loop that normally occur only in endosomes as a first step of low‐pH‐triggered viral membrane fusion (Fig. 2B).

Of note, FcγR‐independent enhancement of infection was also observed with another flavivirus (West Nile virus) and a mab (E100) that, like mab A5, binds to an epitope at a similar site in domain II at the E dimer interface [75]. Based on this similarity, the mechanism of increased infectivity may be analogous and involve antibody‐promoted E dimer dissociation in both instances. Since mabs A5 and E100 enhance infection, they are probably unable to block fusion in the endosome, even after co‐internalization with the virus. Post‐entry neutralization would require virus‐antibody interactions that are resistant to acidic pH. Since the epitopes of mab A5 and mab E100 each involve a histidine [75, 76, 77], their binding might be pH sensitive [78, 79] and it is possible (but not experimentally shown) that their protonation in the acidic endosome weakens or even abolishes antibody interactions with E. Structural changes of E required for membrane fusion (Fig. 2) would thus not be blocked by these antibodies, allowing infection to proceed unimpaired.

## Structural dynamics and formation of the E post‐fusion structure

**Fig. 6:**
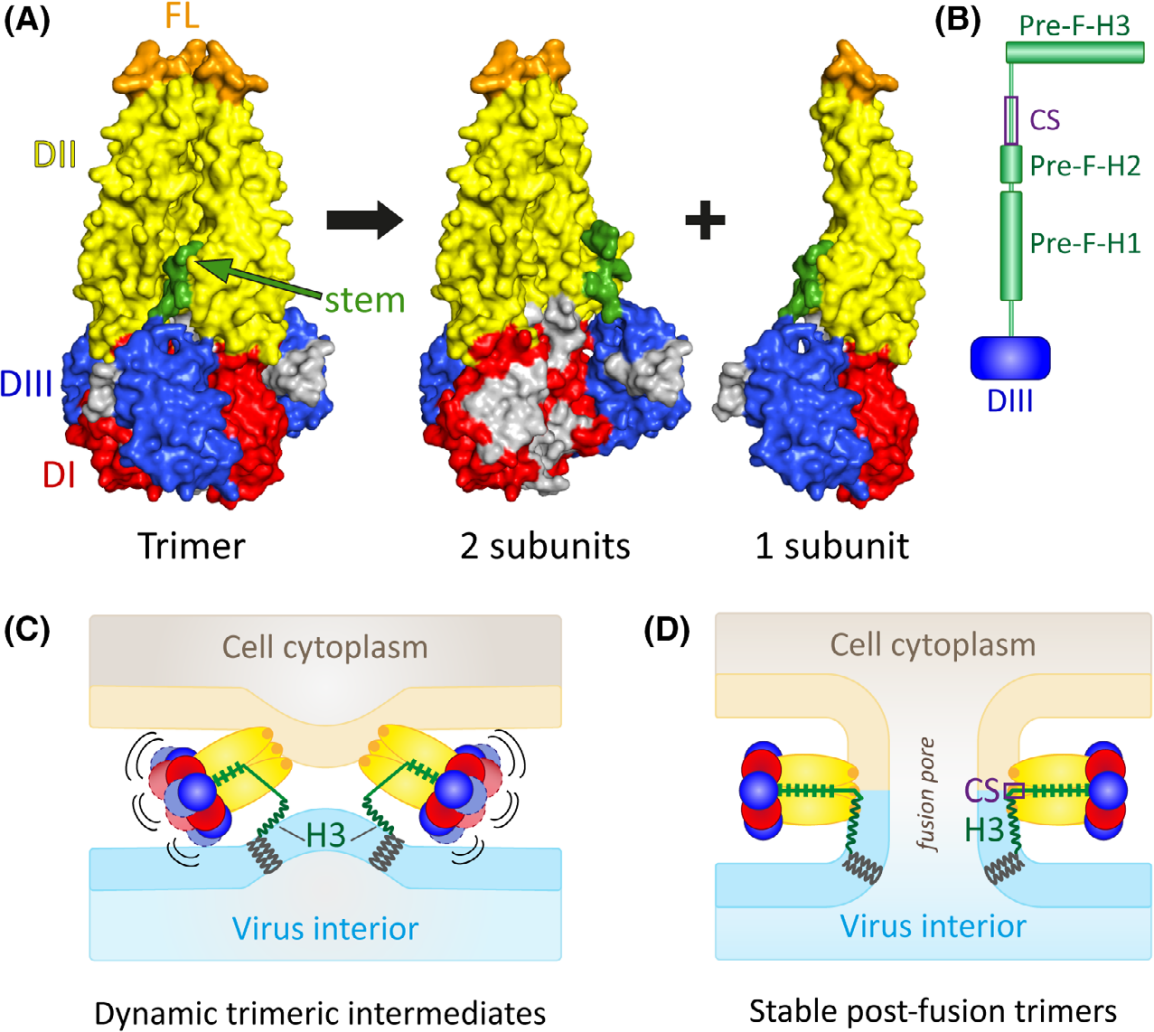
Dynamic trimeric fusion intermediates in flavivirus membrane fusion. (A) Side view of the E protein trimer (left), and open book view of two subunits and the missing “open” subunit. The dynamically exposed regions in fusion intermediates are shown in grey. (B) Schematic of the pre‐fusion organization of the E stem based on cryo‐EM of TBEV [19]. Pre‐F‐H1, stem helix 1 in its pre‐fusion conformation; Pre‐F‐H2, stem helix 2 in its pre‐fusion conformation; (Pre‐F‐) H3, stem helix 3 in pre‐ and post‐fusion form; CS, conserved sequence. (C) Dynamic intermediate after relocation of domain III with incompletely zippered stem (“breathing trimers”). (D) Final post‐fusion trimer with the fully zippered stem and formation of the fusion pore. Only one of the three stem‐anchor regions in the trimer is shown in panels (C) and (D). Colour code E as in Figs. 1 and 2. Molecular structures shown in panel A were generated with PyMol (https://pymol.org/2) using coordinates of TBEV (PDBs: 1URZ, 6S8C [27, 31]).

Protein dynamics were not only shown to play an important role in the interaction of flaviviruses with antibodies and virus‐cell interactions, but also in the conformational changes of E required for mediating membrane fusion [31]. The mechanism of flavivirus membrane fusion schematically depicted in Fig. 2 was inferred from biochemical studies [24, 25, 80, 81, 82, 83] and crystal structures of E proteins of several flaviviruses in their trimeric post‐fusion conformations [26, 27, 28, 29, 30, 31] (Fig. 6A). These structures, however, lacked the stem‐anchor regions that are essential for driving the merger of viral and cellular membranes (Fig. 2).

Recent work with TBEV E post‐fusion trimers containing stem extensions of different lengths provides evidence for highly dynamic intermediates of E in the membrane fusion process [31]. The stem extensions corresponded to the pre‐fusion (Pre‐F) organization of this region with three helices (Pre‐F‐H1, Pre‐F‐H2, Pre‐F‐H3) and a conserved sequence (CS) element between Pre‐F‐H2 and Pre‐F‐H3 [19] (Fig. 6B). Based on the obtained data, the flavivirus fusion mechanism is therefore believed to proceed as follows: The first stages of the process include fusion‐loop exposure and insertion into the target membrane as well as initiation of hairpin formation through the relocation of DIII from the end to the side of the molecule (Fig. 2). Subsequently, a few N‐terminal Pre‐F‐H1 residues of the stem start to zipper along the trimer core (Fig. 6C), anchoring the N‐terminal end of the stem in a groove formed by domains II in the trimer. This intermediate stage is highly dynamic (“breathing trimer”), in which the incompletely stem‐zippered E trimers sample a broad conformational landscape (Fig. 6C) and transiently expose residues of the trimer interface (in particular in domains I and III, Fig. 6A). The C‐terminal helix (Pre‐F‐H3) of the stem is then hypothesized to pull toward domain II along with the membrane anchor, and the N‐terminal part of the stem (Pre‐F‐H1+Pre‐F‐H2+CS) continues zippering up to reach the fusion loops (Fig. 6C,D), leading to membrane merger. The asymmetry of the zippering reaction relative to the target membrane and a tilting of the trimer at this stage has been hypothesized to be crucial for executing membrane merger [31]. In the stable final post‐fusion trimer, it is assumed that the N‐terminal pre‐fusion helices H1 and H2 have lost their helical structure and are fully zippered along domain II, with the CS of the stem interacting with the fusion loop and Pre‐F‐H3 remaining bound to the fused membrane as a helix [31] (Fig. 6D).

The experiments with TBEV have thus identified a new stage of the flavivirus life cycle in which E protein “breathing” appears to be essential for allowing the molecular gymnastics required for membrane fusion in the endosome. These findings extend the importance of envelope protein dynamics to processes beyond those occurring at the viral surface.

## Perspectives and open questions

High‐resolution cryo‐EM structures of flavivirus particles at different stages of virus maturation as well as X‐ray structures of individual proteins and protein complexes have provided unprecedented structural insights into the molecular biology of these viruses [11, 69, 84]. This holds especially true for the mechanisms of viral entry, including receptor interactions [12, 21] as well as the molecular acrobatics of E required for viral membrane fusion [16, 31], its triggering by acidic pH in the endosome and how premature fusion during exocytosis is prevented by specific oligomeric interactions in immature particles [37, 39, 85]. The extensive structural knowledge allowed a detailed characterization of the antigenic landscape of flaviviruses, defining a spectrum of epitopes involved in the induction of antibodies in infected and vaccinated hosts, and elucidating the range of mechanisms leading to virus neutralization as well as protection [13, 14]. The identification of flavivirus “breathing” through studies with antibodies [15] added another layer of complexity to the static pictures obtained by structural biology techniques. Apparently, the proteins at the viral surface are in dynamic motion and can sample different conformations at equilibrium, thus exposing sites that would otherwise be buried through protein‐protein interactions [13, 14]. Of note, the strength of interactions between E monomers in E dimers appears to differ among flaviviruses [61, 86, 87], suggesting that also the extent of breathing and/or ligand‐induced exposure of cryptic reactive sites may vary. It can be hypothesized that such differences reflect adaptations to the specific requirements of the natural cycles of these viruses.

**Fig. 7:**
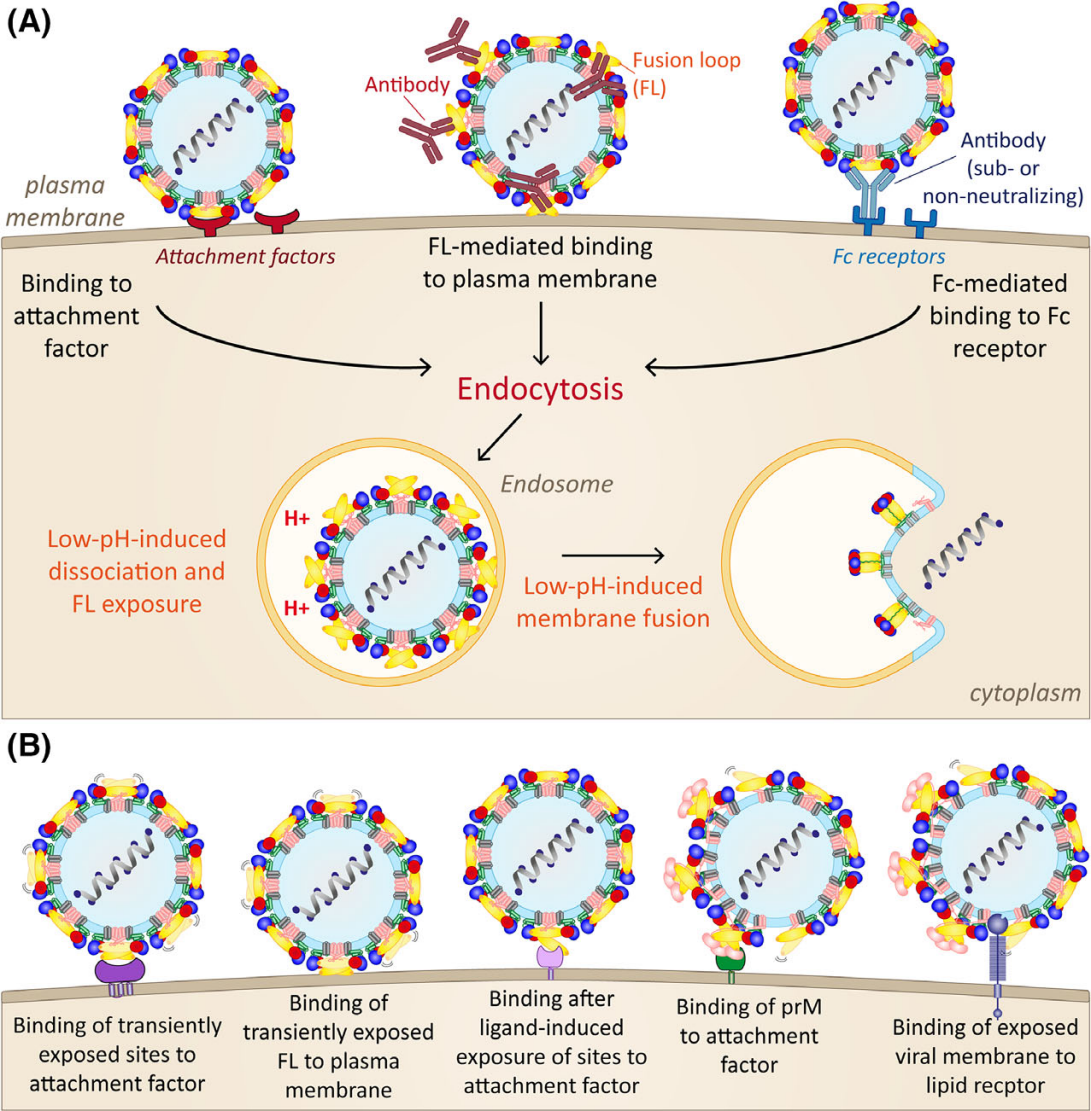
Schematic representation of different modes of cell attachment. (A) Known cell‐binding modes of flaviviruses. Left panel: after binding to a specific receptor. Middle panel: antibody‐induced exposure of the FL and attachment to the plasma membrane. Right panel: FcR‐mediated uptake of an antibody‐virus complex. (B) Hypothetical cell‐binding modes of dynamic and/or heterogeneous flavivirus particles. Colour code E: DI, red; DII, yellow; DIII, blue; fusion loop (FL), orange; stem, green and membrane anchor, grey. The (pr)M protein is shown in pink.

Although the relevance of virus breathing is now clearly documented for virus‐antibody interactions, its impact may go far beyond this immunological aspect and extend more generally to the multitude of virus‐host interactions in the ecological cycles of flaviviruses. A number of molecules have been described as potential flavivirus receptors in vitro [21, 88], but the situation in natural hosts might be more complex, because the maintenance of these viruses in nature depends on their replication in evolutionarily very distant host environments [9, 89, 90]. Protein dynamics at the viral surface, combined with the formation of infectious particles that are only partially mature can amplify the surfaces available for host cell binding, thus increasing the opportunities for efficient virus‐cell interactions. In addition, binding of cellular ligands to viral envelope proteins may shift the equilibrium of protein conformations (as shown by antibody binding in our experiments with TBEV [71]) and, as a consequence, may specifically control virus‐cell interactions in relevant tissues of different hosts. Some of these scenarios are depicted in Fig. 7 and require further investigation.

Considering the fact that single amino acid exchanges can have a profound effect on E dynamics [51, 52, 54], one might speculate that mutational modulations of the extent of breathing contributed to the evolution of extant flaviviruses and the adaptation to their arthropod and vertebrate hosts. In general, flaviviruses replicate at temperatures ranging from 37 °C up to 44 °C in their vertebrate hosts, then switch to their ectotherm vectors, in which temperature variations depend on the outside temperature [90]. These dramatic temperature changes can affect not only virus replication but also breathing of virions in the different hosts, favouring the selection of temperature‐adapted virus variants. In this context, it is of note that TBEV (occurring in temperate climate zones) appears to be more stable than dengue viruses (circulating in tropical and subtropical regions) [61], and a recent study provides evidence that tick‐borne flaviviruses are ancestral to their mosquito‐borne counterparts [91]. The capacity of more or less extensive breathing at higher temperatures [15] may also be relevant for the adaptation of several flaviviruses (e.g. West Nile, Japanese encephalitis and Usutu viruses) for replication in birds, which have a higher body temperature than mammalian hosts [92]. All of these aspects are rich in unresolved scientific questions and provide ample opportunities for future research towards a deeper understanding of flavivirus evolution and the biological complexity of flavivirus replication in their natural hosts.

## Conflict of interest

None to declare with this review. The Center for Virology received a research grant from Pfizer on the epidemiology of TBE in Austria, with KS as principal investigator (2018‐2021). FXH and KS are inventors on a patent of the Medical University of Vienna on flavivirus IgM serodiagnosis.

## Author contributions

KS and FXH wrote the manuscript. IM and LR created the structural representations for Fig. 1 and 6. KS generated the figures. KS, IM and LR performed literature search. All authors were involved in figure design and editing the article.

## Data Availability

The PDB codes of the coordinates used for generating the structural representations in Figures 1, 2, 3, 5 and 6 are cited in the corresponding legends.
